# Supplementary material for: Early Identification of Hospital Visit Risk in Heart Failure Using Wearable-Derived Data
Source: medRxiv. 2026 Mar 27:2026.03.26.26349411. Preprint. [Version 1] doi: 10.64898/2026.03.26.26349411 (PMC13042084; doi:10.64898/2026.03.26.26349411)
Supplement: Supplement 1 [file NIHPP2026.03.26.26349411v1-supplement-1.pdf]

medRxiv preprint doi: https://doi.org/10.64898/2026.03.26.26349411; this version posted March 27, 2026. The copyright holder for this preprint (which was not certified by peer review) is the author/funder, who has granted medRxiv a license to display the preprint in perpetuity.
It is made available under a CC-BY 4.0 International license .

SUPPLEMENTARY MATERIALS

Visit count differences between groups

A statistically significant difference was found in visit counts between the group with no app and those with an app (see Table S3). Figure S1 shows survival curves for each study group with group D having a sharper decline.

Study Group	Participant Count	Participants with Visits	Total Visits
D	72	22 (25%)	34
D+A	89	15 (17%)	18
D+A+F	88	14 (16%)	28
**Total**	**249**	**51 (20%)**	**80**

TABLE S3. **Study Group Counts:** Total number of participants and visits across study groups.

Addition of an app does not impact trends in steps

To ensure the use of an app was not a confounding variable in the step count analysis, we evaluated step survival curves stratified by group. Since both group D+A and D+A+F used apps we combined them in this analysis. Step count distribution during the baseline period of two weeks were not significantly different between groups ($p=.67$).

medRxiv preprint doi: https://doi.org/10.64898/2026.03.26.26349411; this version posted March 27, 2026. The copyright holder for this preprint (which was not certified by peer review) is the author/funder, who has granted medRxiv a license to display the preprint in perpetuity.
It is made available under a CC-BY 4.0 International license .

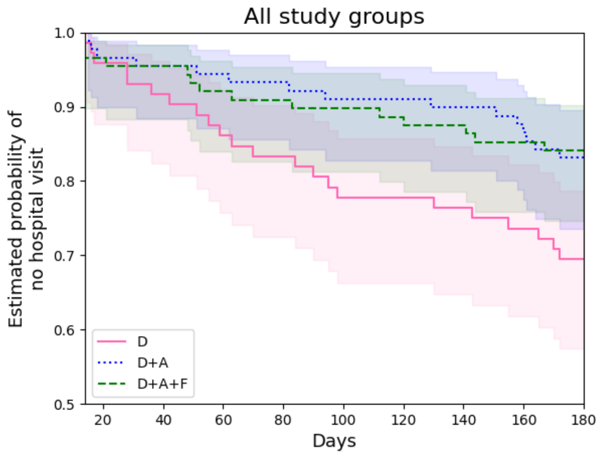

FIG. S1. **Kaplan-Meier curves by study group:** Group D had significantly more visits than both Groups D+A and D+A+F.

medRxiv preprint doi: https://doi.org/10.64898/2026.03.26.26349411; this version posted March 27, 2026. The copyright holder for this preprint (which was not certified by peer review) is the author/funder, who has granted medRxiv a license to display the preprint in perpetuity.
It is made available under a CC-BY 4.0 International license .

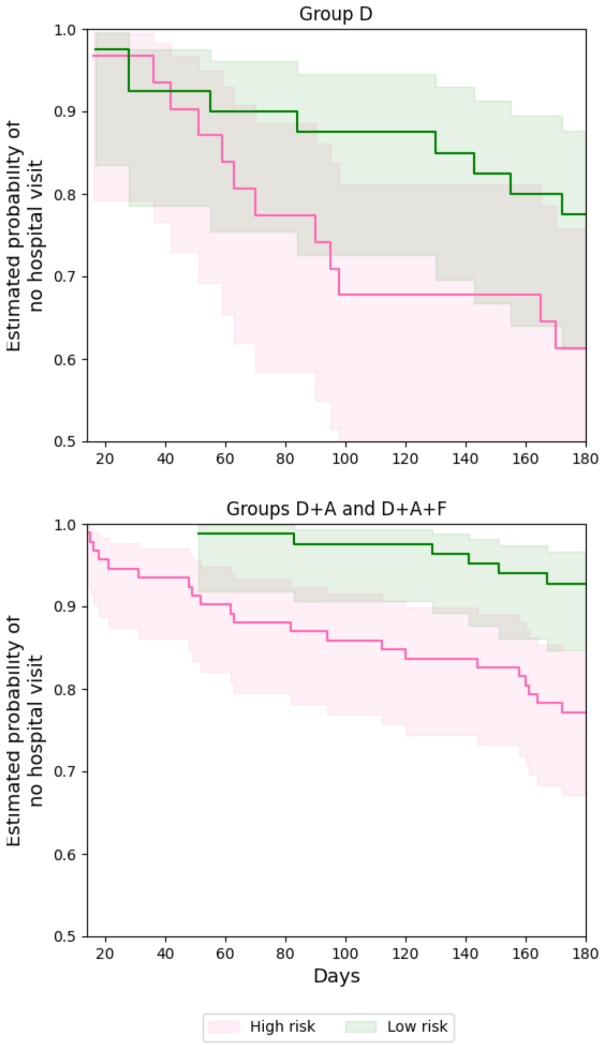

FIG. S2. **Group stratified Kaplan-Meier step curves:** Steps were a predictor of hospital visits for all study group regardless of having an app.
